# Antimicrobial stewardship programs and convalescent plasma for COVID-19: A new paradigm for preauthorization?

**DOI:** 10.1017/ice.2020.459

**Published:** 2020-09-09

**Authors:** Michael P. Stevens, Payal K. Patel, Priya Nori

**Affiliations:** 1 Healthcare Infection Prevention Department, Virginia Commonwealth University Health System, Richmond, Virginia; 2 Infectious Diseases Section, Ann Arbor VA Medical Center, Ann Arbor, Michigan; 3 University of Michigan, Ann Arbor, Michigan; 4 Division of Infectious Diseases, Department of Medicine, Montefiore Medical Center, Albert Einstein College of Medicine, Bronx, New York

*To the Editor—*Antimicrobial preauthorization is a core strategy utilized by antimicrobial stewardship programs (ASPs).^1^ ASPs have played an important role in coronavirus disease 2019 (COVID-19) response efforts, including in the preauthorization of novel therapeutic agents such as remdesivir.^2,3^ On August 23, 2020, the US Food & Drug Administration (FDA) released an emergency use authorization (EUA) for the use of convalescent plasma in treating hospitalized patients with COVID-19.^4^ An important question is what role, if any, ASPs should play in the convalescent plasma distribution process. To our knowledge, ASPs have never been involved in the preauthorization of blood products like convalescent plasma. There are numerous potential advantages and disadvantages to consider regarding ASP involvement in the convalescent plasma preauthorization process (Table 1). The effectiveness of convalescent plasma in the treatment of COVID-19 is still unclear. The data regarding convalescent plasma use are limited. As of June 22, 2020, the Infectious Diseases Society of America (IDSA) COVID-19 treatment guidelines recommend the use of convalescent plasma only in the context of a clinical trial.^5^ Importantly, enrollment in existing trials has been potentially compromised by the EUA announcement. Major scientific organizations will likely continue to support guidelines emphasizing convalescent plasma use only in the context of clinical trials. It is also possible that additional study data will become available that will influence convalescent plasma use. This uncertainty about the optimal role of convalescent plasma supports the use of preauthorization to allow for real-time adjustment of convalescent plasma use in a controlled, optimized fashion.

**Table 1.** Considerations For and Against Antimicrobial Stewardship Program (ASP) Involvement in COVID-19 Convalescent Plasma Preauthorization

For ASP Involvement	Against ASP Involvement
• ASPs already have preauthorization infrastructure in place • Transfusion medicine programs likely would need to create pre-authorization processes de novo and identify how to staff these • ASP personnel are experts at creating and applying algorithm-based preauthorization criteria • ASPs that are already responsible for local COVID-19 guidelines can help contextualize convalescent plasma use relative to other potential therapies • ASP personnel are experts at cooperative integration with non–infectious diseases or non–pharmacy-based service lines	• ASPs have no direct involvement with transfusion medicine programs or authority to restrict access to blood products • ASP personnel are not experts in transfusion medicine • ASP involvement will divert time away from other important stewardship activities, such as antibiotic use monitoring • ASPs are put in the difficult position of brokering convalescent plasma access against scientific community recommendations to use only in the context of randomized, clinical trials

Many ASPs have been responsible for the creation and maintenance of COVID-19 treatment guidelines and are ideally situated to inform frontline clinicians about the optimal use of convalescent plasma relative to other therapies. Preauthorization, coupled with local treatment guidelines, would enhance the optimal use of convalescent plasma. Additionally, the new convalescent plasma EUA may increase demand for convalescent plasma use, resulting in timely access issues. A preauthorization process utilizing the best available evidence would facilitate providing convalescent plasma to patients who may benefit.

Health systems would benefit tremendously from ASP involvement in the COVID-19 convalescent plasma distribution process. ASPs can provide guidance for incorporation of convalescent plasma into local treatment guidelines, can provide insight and guidance based on their experiences with other COVID-19 focused EUAs (including hydroxychloroquine, now revoked^6^, and remdesivir^7^), and can help develop processes for convalescent plasma eligibility screening and preauthorization. If health systems do not adopt preauthorization for convalescent plasma, we recommend that use be carefully monitored to ensure that this resource is being used optimally. ASPs have proven integral in COVID-19 response efforts—investing in and scaling up ASP resources will assist health systems adapt and respond to evolving pandemic challenges.
